# Rare dual MYH9–ROS1 fusion variants in a patient with lung adenocarcinoma: A case report

**DOI:** 10.1097/MD.0000000000041350

**Published:** 2025-01-24

**Authors:** Tian Luo, Wentao Ji, Weihong Guo, Dandan Zhang, Jianping Liang, Yanhua Lv

**Affiliations:** a Zhongshan City People’s Hospital, Xinxiang Medical University, Xinxiang, Henan, China; b Department of Respiratory and Critical Care Medicine, Zhongshan City People’s Hospital, Zhongshan, Guangdong Province, China.

**Keywords:** case report, crizotinib, lorlatinib, lung adenocarcinoma, MYH9–ROS1, non-small cell lung cancer, TP53

## Abstract

**Rationale:**

ROS proto-oncogene 1 (ROS1) fusion is a rare but important driver mutation in non-small cell lung cancer, which usually shows significant sensitivity to small molecule tyrosine kinase inhibitors. With the widespread application of next-generation sequencing (NGS), more fusions and co-mutations of ROS1 have been discovered. Non-muscle myosin heavy chain 9 (MYH9) is a rare fusion partner of ROS1 gene as reported. Here, we report an even rare case with coexistence of short and long variants MYH9–ROS1 fusions at the RNA level accompanied by TP53 mutation, insensitively to antitumor therapy.

**Patient concerns and diagnosis:**

A 37-year-old nonsmoking man was diagnosed with stage IVB (T4N3M1c) lung adenocarcinoma. The tumor was identified to have MYH9 (exon 37)–ROS1 (exon 35) rearrangement with TP53 mutation at the DNA level by DNA-NGS analysis of lymph node biopsy tissue in March 2023. Interestingly, it was transcribed into coexistence of short and long variants MYH9–ROS1 (M36, R36) and MYH9–ROS1 (M36, R35) fusions at RNA level by RNA-NGS analysis.

**Interventions:**

First-line tyrosine kinase inhibitors crizotinib was given firstly, showing partial response (PR) but significant progression within 3 months. To determine the resistance mechanism to crizotinib and the genetic variation, DNA-NGS and RNA-NGS were performed again on a new biopsy tissue of lymph node in August 2023.

**Outcomes:**

Rare coexistence of short and long variants of MYH9–ROS1 fusions was identified again, but the typical mechanisms of crizotinib resistance were not observed. Switching to lorlatinib resulted in brief PR about 2 months. Subsequent 2 courses of system chemotherapy provided short-term PR less than 2 months. The patient died with a total survival of 10 months.

**Lessons:**

We must pay attention to rare dual short and long variants of the MYH9–ROS1 fusions, it may affect the efficacy of ROS1-tyrosine kinase inhibitors targeted therapy.

## 1. Introduction

Lung cancer is one of the leading malignancies worldwide. The ROS proto-oncogene 1 (ROS1) gene rearrangement has been identified in 1% to 2% of non-small cell lung cancer (NSCLC) patients.^[1]^ To date, the known fusion partners of ROS1 include CD74, SDC4, and EZR.^[2]^ With the increasing application of next-generation sequencing (NGS), more rare fusion genes have been identified, and these complex fusions and mutations may impact the effectiveness of drug therapy. Among them, non-muscle myosin heavy chain 9 (MYH9) is a rare fusion partner of ROS1, accounting for approximately 1% of ROS1 cases. In this study, we report a rare case that coexistence of short and long variants MYH9–ROS1 (M36, R36) and MYH9–ROS1 (M36, R35) mutations at RNA level. The patient was insensitively to antitumor therapy, including crizotinib, lorlatinib, and chemotherapy.

## 2. Case presentation

**Figure 1.**
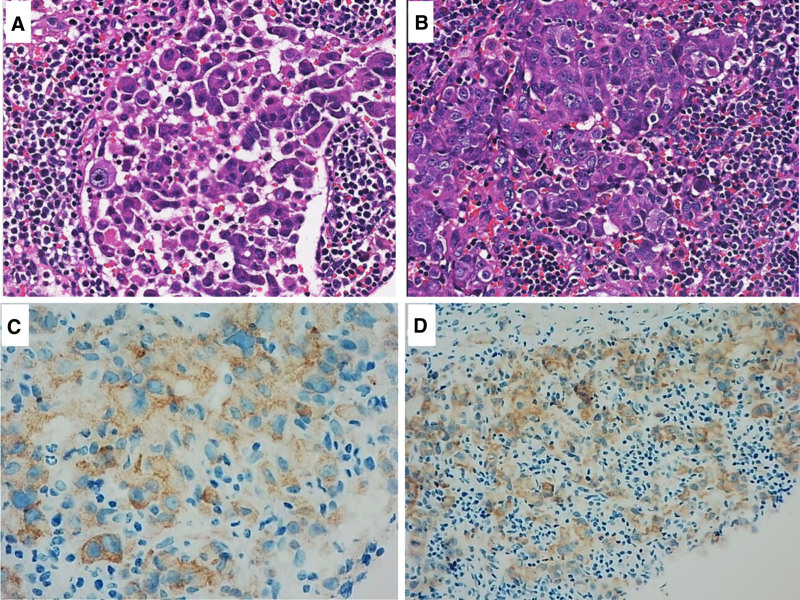
(A and B) Histopathological images of lymph node biopsy samples from the patient; (C and D) ROS1 (1:400, GeneTech, Shanghai, Co., Ltd, China) immunohistochemistry staining.

**Figure 2.**
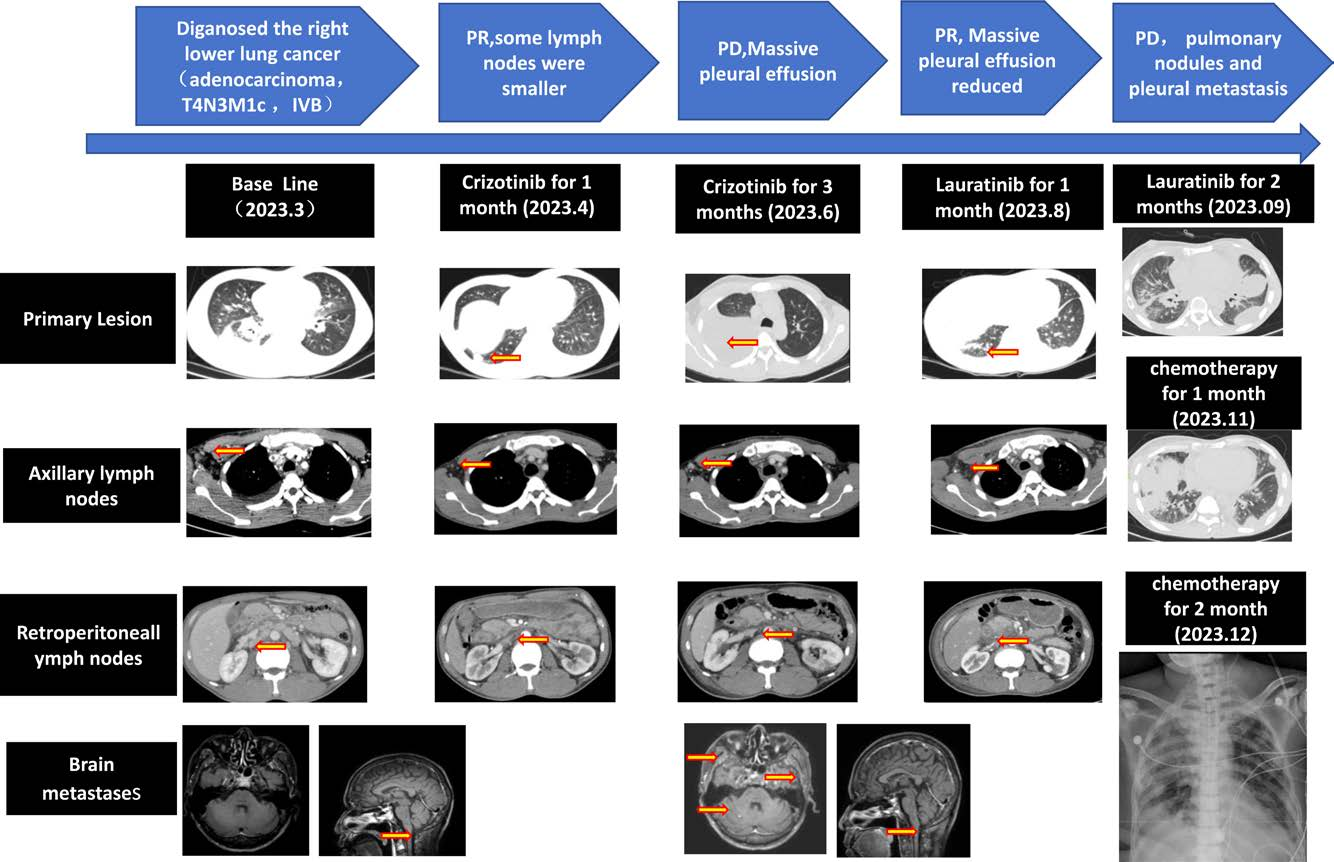
Timeline of clinical treatment course and radiologic feature.

The patient, a 37-year-old man with no history of smoking and no family history of cancers, was admitted to the hospital in March 2023 due to “cervical lymphadenopathy for 1 week.” During hospitalization, a right cervical lymph node biopsy was obtained for postoperative pathology, which revealed metastatic adenocarcinoma of the lung (Fig. 1). In addition, images from enhanced CT of the whole abdomen, enhanced MR Of the skull, and whole-body bone ECT suggested a right lower lung cancer with multiple enlarged lymph nodes in the bilateral neck, hilum mediastinum, axilla, abdominal cavity, and retroperitoneal regions. The patient was diagnosed with right lower lung cancer (adenocarcinoma, T4N3M1c, stage IVB) (Fig. 2). DNA-NGS and RNA-NGS results of lymph node tissue revealed a positive MYH9–ROS1 fusion. ROS1 rearrangement is positive by immunohistochemical staining using antibody against ROS1 (1:400, Gene Tech Company Limited, China). Crizotinib (0.25 g BID) was chosen for first-line antitumor therapy in accordance with the 2023 NCCN guidelines. After 1 month, the enlarged lymph nodes in the bilateral neck, mediastinal hilum, axilla, abdominal cavity, and retroperitoneal regions were significantly reduced, with no significant enlargement of the primary lesion in the right lower lung (Fig. 2). After 3 months of the therapy, the patient was readmission due to dyspnea. Multiple nodules in the anterior mediastinum, moderate pleural effusion in the right thoracic cavity, and multiple new nodules on the right lower pleura were revealed. In addition, new metastatic foci in the bilateral cerebral and cerebellar hemispheres were found, considered as Trousseau syndrome (Fig. 2). This suggests widespread systemic progression of the tumor that signified the acquisition of crizotinib resistance.

**Figure 3.**
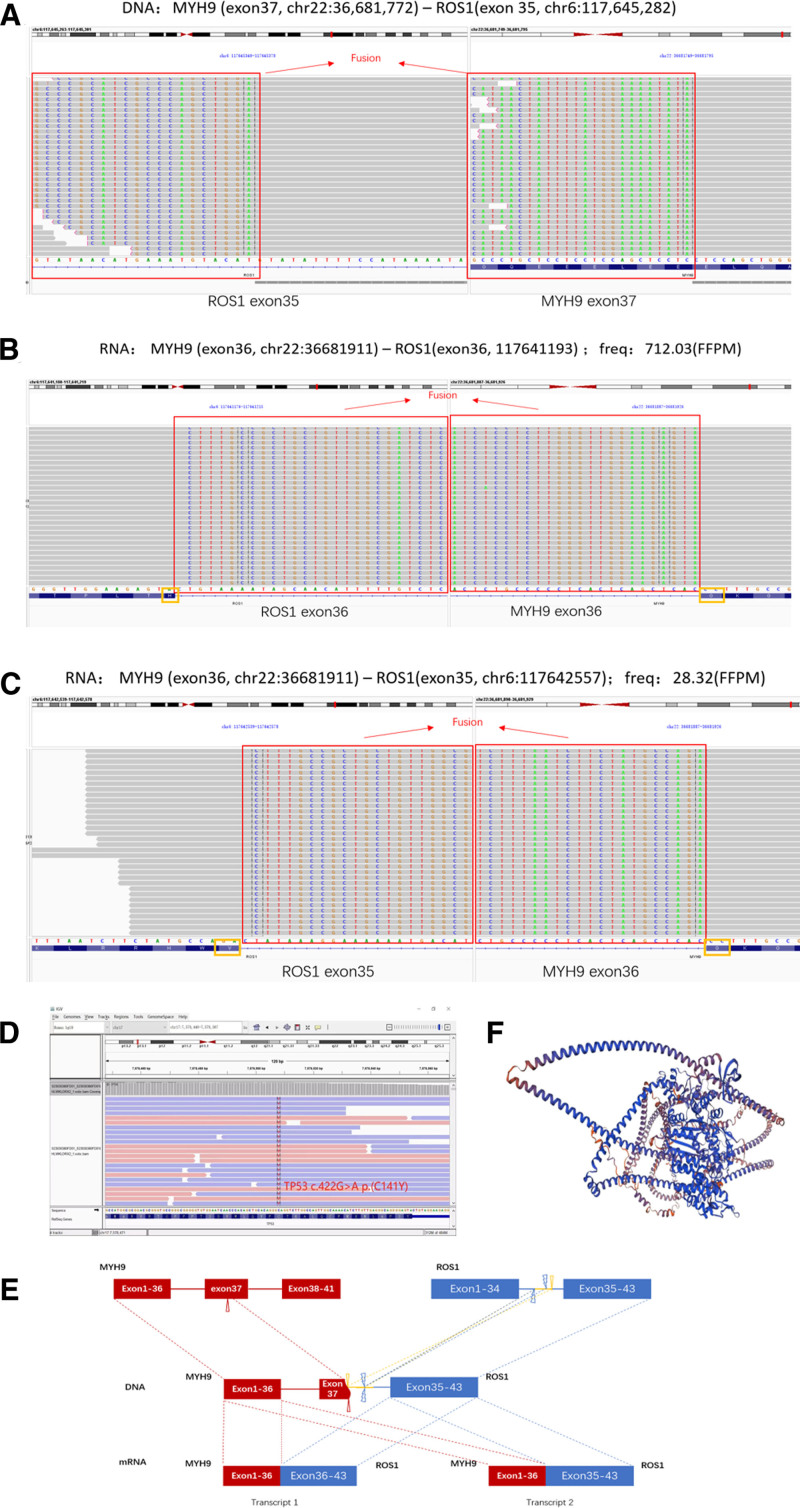
Identification and verification of MYH9–ROS1 fusion. (A) DNA-NGS MYH9 (exon 37)–ROS1 (exon 35). (B) RNA-NGS MYH9–ROS1 (M36, R35) from the first biopsy in March, 2023. (C) RNA-NGS MYH9–ROS1 (M36, R35) from the second biopsy in August, 2023. (D) DNA-NGS TP53 co-mutation from the first biopsy in March, 2023. (E) Schematic of genomic rearrangement. (F) Fusion regions were visualized by the Integrative Genomic Viewer (IGV) software.

In order to determine if there is a genetic variation causing crizotinib resistance, DNA-NGS and RNA-NGS were performed again on a new biopsy tissue of lymph node after drug resistance of the patient on August 1, 2023. We compared the results of the 2 genetic tests and found that the results were consistent in the fusion patterns of MYH9 (exon 37)–ROS1 (exon 35) at the DNA level (Fig. 3), accompanied by a co-mutation of TP53 (Fig. 3). However, at the RNA level, the patient presented rare coexistence of short and long variants, MYH9 (exon 1–36)–ROS1 (exon 36–41) and MYH9 (exon 1–36)–ROS1 (exon 35–41), which were abbreviated as MYH9–ROS1 (M36, R36) and MYH9–ROS1 (M36, R35) (Fig. 3). IGV software analysis showed that MYH9–ROS1 (M36, R36) appeared as an in-frame fusion and MYH9–ROS1 (M36, R35) as out-of-frame. However, the typical mechanisms of crizotinib resistance reported in the current literature, such as G2032R/K, D2033N, S1986Y/F, 22026M, and L1951R mutations^[3]^ were not found in the second biopsy.

Lorlatinib (100 mg QD) was prescribed for second-line antitumor therapy in accordance with the 2023 NCCN guidelines. Pleural effusion and primary lesion were reduced, indicating that lorlatinib is effective, but the progression-free survival (PFS) is only 2 months. Subsequent 2 courses of system chemotherapy with pemetrexed-carboplatin provided short-term partial response less than 2 months. The patient died with tumor progression and leukemoid reaction in December, 2023 with a total survival of ten months.

Written informed consent was obtained from the patient’s wife.

## 3. Discussion

ROS1 fusion is a rare but important driver mutation in NSCLC, which usually shows significant sensitivity to small molecule tyrosine kinase inhibitors (TKIs), such as crizotinib, alectinib, entrutinib, ceritinib, lorlatinib, and brigatinib.^[4]^ Since the various fusion variants may have an effect on therapeutic efficacy, accurate detection of ROS1 rearrangements is critical.

This case presents a rare fusion of MYH9–ROS1 that, more rarely, contained both short and long fusion variants. The NCCN2023 guidelines consider crizotinib and entrutinib as the preliminary options for the targeted treatment of ROS1-positive NSCLC at stage IV.^[4]^ According to available reports, the median progression-free survival (mPFS) of ROS1-positive patients treated with crizotinib was 20.0 months.^[5]^ However, this patient developed resistance to crizotinib within only 3 months, and the typical mechanisms of crizotinib resistance that have been reported were not observed in the second biopsy, such as G2032R/K, D2033N, S1986Y/F, 22026M, L1951R mutation, etc. Lorlatinib was given for the second-line targeted treatment. A multicentre, open-label, single-arm, phase 1 to 2 trial (NCT01970865) showed that the activity of lorlatinib was notably lower in crizotinib-pretreated patients than in TKI-naive patients, with 35% of patients achieving an objective response and mPFS of 8.5 months.^[6]^ Unfortunately, the patient’s PFS was only 2 months after receiving treatment with lorlatinib.

Therefore, it is necessary to discuss the drug resistance mechanism of this patient. On the one hand, the presence of TP53 co-mutations was noted in this patient. Studies have confirmed that coexistence of TP53 affects the efficacy of EGFR, ALK, and ROS1 TKIs therapy as determined by relatively short mPFS.^[7]^ Moreover, ROS1 and TP53 co-mutation also shorten the mPFS of the patients with lung cancer as reported.^[5,8]^ Therefore, we speculated that the patient’s resistance to crizotinib may be related to the coexistence of TP53 mutations. However, according to the relevant literature, even with poor efficacy, patients with TP53 mutations experienced mPFS longer than 7 months after crizotinib treatment,^[5,7,8]^ but the PFS of this patient with crizotinib treatment was <3 months, as well as lorlatinib.^[9]^ Therefore, we speculate that TP53 mutations may not be the principal cause of crizotinib and lorlatinib tolerance in this patient.

This patient is a rare case of coexistence of MYH9–ROS1 (M36, R36) and (M36, R35). It has been reported that ALK and its ligands break and fused as various forms that can generate short and long variants and complex fusions. For example, the common EML4–ALK fusions includes multiple subtypes such as EML4–ALK V1 (E13, A20), V2 (E20, A20), V3a/b (E6, A20), and EML4–ALK V1 (E13, A20). Moreover, The mPFS of patients with long variants treated with crizotinib is 10.9 months, while the mPFS of patients with short variants is only 2.2 months, so patients with different short and long variants respond differentially to ALK-TKI therapy.^[10,11]^ There are also different breakpoints in ROS1 fusion. ROS1 contains 43 exons, and the common breakpoints of ROS1 are exons 34, 35, and 36.^[12]^ However, few studies have reported the coexistence of 2 breakpoint fusions in the same patient. The ROS1 fusion of this patient resulted from breakpoints at 35 and 36. The complex fusions of MYH9–ROS1 may be the main reason for the rapid acquisition of crizotinib resistance. IGV software analysis revealed that MYH9–ROS1 (M36, R36) was an in-frame fusion, while MYH9–ROS1 (M36, R35) was an out-frame fusion. Rather than out-frame fusions, sn-frame fused transcripts are thought to express a functionally activated fusion protein and maintain sensitivity to ROS1 inhibitors. Since the out-frame fusion RNAs may act like long noncoding RNAs to exert regulatory functions,^[13,14]^ we speculate that the out-frame fusion of MYH9–ROS1 (M36, R35) may be responsible for the rapid resistance to crizotinib and lorlatinib. However, the speculation requires more documentation and investigation on the coexistence of 2 fusion variants of MYH9–ROS1.

## 4. Conclusion

In this report, we present a rare case of lung adenocarcinoma with the coexistence of short and long variants of the MYH9–ROS1 fusions, which may affect the efficacy of ROS1-TKI targeted therapy.

## Author contributions

**Conceptualization:** Tian Luo, Yanhua Lv.

**Data curation:** Wentao Ji, Weihong Guo.

**Formal analysis:** Yanhua Lv.

**Investigation:** Weihong Guo.

**Methodology:** Dandan Zhang, Jianping Liang.

**Writing – original draft:** Tian Luo.

**Writing – review & editing:** Weihong Guo, Yanhua Lv.
